# A joint analysis of metabolomics and genetics of breast cancer

**DOI:** 10.1186/s13058-014-0415-9

**Published:** 2014-08-05

**Authors:** Xiaohu Tang, Chao-Chieh Lin, Ivan Spasojevic, Edwin S Iversen, Jen-Tsan Chi, Jeffrey R Marks

**Affiliations:** 1 Department of Molecular Genetics and Microbiology, Duke University, 268 CARL Building, Research Drive, Durham, 27708 NC USA; 2 Duke Center for Genomic and Computational Biology, Duke University, 101 Science Drive, Durham, 27708 NC USA; 3 Department of Medicine, Duke University, 201 Trent Drive, Durham, 27710 NC USA; 4 Department of Statistical Science, Duke University, 214A Old Chemistry Building, Durham, 27710 NC USA; 5 Department of Surgery, Division of Surgical Sciences, Duke University, 103 Research Drive, Durham, 27710 NC USA

## Abstract

**Introduction:**

Remodeling of cellular metabolism appears to be a consequence and possibly a cause of oncogenic transformation in human cancers. Specific aspects of altered tumor metabolism may be amenable to therapeutic intervention and could be coordinated with other targeted therapies. In breast cancer, the genetic landscape has been defined most comprehensively in efforts such as The Cancer Genome Atlas (TCGA). However, little is known about how alterations of tumor metabolism correlate with this landscape.

**Methods:**

In total 25 cancers (23 fully analyzed by TCGA) and 5 normal breast specimens were analyzed by gas chromatography/mass spectrometry and liquid chromatography/mass spectrometry, quantitating 399 identifiable metabolites.

**Results:**

We found strong differences correlated with hormone receptor status with 18% of the metabolites elevated in estrogen receptor negative (ER-) cancers compared to estrogen receptor positive (ER+) including many glycolytic and glycogenolytic intermediates consistent with increased Warburg effects. Glutathione (GSH) pathway components were also elevated in ER- tumors consistent with an increased requirement for handling higher levels of oxidative stress. Additionally, ER- tumors had high levels of the oncometabolite 2-hydroxyglutarate (2-HG) and the immunomodulatory tryptophan metabolite kynurenine. Kynurenine levels were correlated with the expression of tryptophan-degrading enzyme (*IDO1*). However, high levels of 2-HG were not associated with somatic mutations or expression levels of *IDH1* or *IDH2. BRCA1* mRNA levels were positively associated with coenzyme A, acetyl coenzyme A, and GSH and negatively associated with multiple lipid species, supporting the regulation of *ACC1* and *NRF2* by BRCA1. Different driver mutations were associated with distinct patterns of specific metabolites, such as lower levels of several lipid-glycerophosphocholines in tumors with mutated *TP53*. A strong metabolomic signature associated with proliferation rate was also observed; the metabolites in this signature overlap broadly with metabolites that define ER status as receptor status and proliferation rate were correlated.

**Conclusions:**

The addition of metabolomic profiles to the public domain TCGA dataset provides an important new tool for discovery and hypothesis testing of the genetic regulation of tumor metabolism. Particular sets of metabolites may reveal insights into the metabolic dysregulation that underlie the heterogeneity of breast cancer.

**Electronic supplementary material:**

The online version of this article (doi:10.1186/s13058-014-0415-9) contains supplementary material, which is available to authorized users.

## Introduction

It is now well established that significant heterogeneity exists among human breast cancers. This heterogeneity is observable at every level of examination from the macroscopic to the molecular. Recent large-scale efforts to measure and describe human breast tumor heterogeneity include The Cancer Genome Atlas (TCGA) where a number of high-throughput ‘omic’ technologies were systemically applied to hundreds of primary cancer specimens [1]. Mutation, germ line polymorphisms, DNA copy number, RNA expression, DNA methylation, and protein expression analyses were performed in parallel on a large and carefully curated set of breast cancer specimens to produce the most comprehensive molecular portrait of the disease to date.

One significant metric that was not included in TGCA was an unbiased analysis of tumor metabolism. While metabolic flux cannot be measured in fixed or frozen specimens, steady-state levels of numerous key metabolites may provide insight into these fundamental phenotypic traits. A number of studies in cancer have uncovered relationships between genetic abnormalities and various metabolic reprogramming suggesting that key metabolic process can be altered as a result of specific transformation events [2]-[6]. Relatively nonspecific cancer-related events such as increased proliferation may also underlie some of the inferred/observed metabolic remodeling. Glucose uptake, serine and glutamine auxotrophy, mitochondrial oxidative phosphorylation, and cancer-associated fibroblasts all appear to have roles in defining breast cancer metabolism [7]-[11]. However, it is not clear whether these regulatory relationships can be observed in all or subsets of human tumors.

Breast cancers are broadly categorized as luminal versus basal types possibly derived from different precursor cells or at least different committed lineages [12]-[14]. Within these broad categories, alterations in specific driver genes are believed to produce the heterogeneity observed amongst and within breast tumor subtypes. While the identity and frequency of driver alterations are generally different in basal and luminal cancers, there is still considerable overlap. For example, TP53 mutations are very common in basal tumors and PI3KCA mutations are common in luminal cancer but neither is subtype exclusive. In contrast, MYC (8q24) amplification is common in both types [1]. Each of these genetic drivers has been associated with specific changes in cellular metabolism and therefore may have dominant effects that can be observed across tumor types.

Metabolomic profiling via mass spectrometry or nuclear magnetic resonance (NMR) is now an established approach that has been employed in several studies to analyze primary human breast tissues (normal and cancer) [15],[16]. Building upon transcriptional profiling of breast cancer, there have also been several efforts to integrate steady-state metabolite levels with specific breast cancer subtypes defined by mRNA expression. Expression subtypes are dominated by estrogen receptor and ERBB2 status and thus, metabolic profiling was performed to seek an additional level of information to refine these existing classifications. These analyses identified a subclassification of luminal A-type cancers based on metabolite levels and found higher levels of Warburg-associated metabolites in more aggressive cancer types [9],[17]. A separate study of breast cancer lipidomic identified the association between palmitate-containing phosphatidylcholines with estrogen receptor negative and cancer progression and patient survival [18]. However, none of these studies established associations of particular metabolites or metabolic pathways with specific somatic mutations or expression levels that have been extensively characterized in TCGA.

In order to more fully explore the relationship between genetics, tumor type, and metabolic state, we took advantage of our participation in the breast cancer TCGA to perform joint analyses of metabolomics and genetics in a series of primary cancers. From the current study, we were able to identify several genetic determinants of the metabolic heterogeneity of human breast tumors that confirm and extend prior *in vitro* and *in vivo* observations.

## Methods

### Specimen selection and handling

Breast tissues were collected, stored and used under Duke University Medical Center Institutional Review Board (IRB) approved protocols (Pro00012025 and Pro00021284). A waiver of consent was obtained from the Duke IRB to conduct the study (Pro00021284) and subjects were not re-consented for participation. Twenty-five breast cancers (diagnosed and treated from 1989 to 1998) were selected for the current study based on their inclusion in The Cancer Genome Atlas (TCGA). Specifically, we selected cases that were either estrogen receptor (ER) positive or negative for both estrogen and progesterone receptors (PR) based on the clinical assay performed at the time of initial diagnosis (clinical and demographic information is provided in Table S1 in Additional file 1). Two of the ER + positive cancers were classified as PR negative by the clinical assay. In addition to the cancers, we selected five breast specimens obtained from reduction mammoplasties containing substantial amounts of normal epithelium. Each block of tissue was cryostat sectioned to analyze tumor epithelial content based on microscopic examination with a cutoff of 70% tumor nuclei for inclusion. Additional sections were also taken and stored desiccated at −80°C for future use. The remainder of the tissue block was submitted frozen to Metabolon Inc. (Durham, NC, USA) for extraction and metabolomic analysis. After trimming away the cryogenic-embedding compound (OCT), the weight of each sample (27 to 115 mg) was determined and used to normalize the extraction reagent volume.

### Proliferation analysis

Thin sections were fixed in acetone and then stained with MIB-1 antibody (Dako, Glostrup, Denmark) that recognizes the Ki-67 proliferation antigen. The mouse monoclonal antibody was used at a final concentration of 200 μg/ml and detected with a biotinylated goat anti-mouse secondary antibody. Following chromogenic detection, each section was scored for the percentage of nuclear-stained epithelial cells. Two hundred epithelial cells were counted in each section spanning at least two high-powered (40X) fields. The proliferation rate was expressed as a percentage of the epithelial cells exhibiting nuclear staining.

### Metabolomic profiling

The sample preparation process at Metabolon was carried out using an automated MicroLab STAR™ system from the Hamilton Company (Reno, NV, USA). Recovery standards were added prior to the first step in the extraction process for quality control purposes. Sample preparation was conducted using a proprietary (Metabolon, Inc.) series of organic and aqueous extractions to remove the protein fraction while allowing maximum recovery of small molecules. The resulting extract was divided into two fractions; one for analysis by liquid chromatography (LC) and one for analysis by gas chromatography (GC). Samples were placed briefly on a Zymark TurboVap (Phoenix Equipment, Inc., Rochester, NY, USA) to remove the organic solvent. Each sample was then frozen and dried under vacuum. Samples were then prepared for the appropriate instrument, either LC/mass spectrometry (MS) or GC/MS.

The LC/MS portion of the platform is based on a Waters ACQUITY UPLC (Waters, Milford, MA, USA) and a Thermo-Finnigan LTQ mass spectrometer (Thermo Fisher Scientific, Waltham, MA, USA), which consists of an electrospray ionization (ESI) source and linear ion trap (LIT) mass analyzer. The sample extract was split into two aliquots, dried, then reconstituted in acidic or basic LC-compatible solvents, each of which contained 11 or more injection standards at fixed concentrations. One aliquot was analyzed using acidic positive ion optimized conditions and the other using basic negative ion optimized conditions in two independent injections using separate dedicated columns. Extracts reconstituted in acidic conditions were gradient eluted using water and methanol both containing 0.1% formic acid, while the basic extracts, which also used water/methanol, contained 6.5 mM ammonium bicarbonate. The MS analysis alternated between MS and data-dependent MS^2^ scans using dynamic exclusion.

The samples destined for GC/MS analysis were redried under vacuum desiccation for a minimum of 24 hrs prior to being derivatized under nitrogen using bis(trimethylsilyl) triflouroacetamide (BSTFA). The GC column was 5% phenyl and the temperature ramp was from 40° to 300°C in a 16 min period. Samples were analyzed on a Thermo-Finnigan Trace DSQ fast-scanning single-quadrupole mass spectrometer using electron impact ionization (Thermo Fisher Scientific). The instrument was tuned and calibrated for mass resolution and mass accuracy on a daily basis. The information output from the raw data files was automatically extracted as discussed below.

For ions with counts greater than 2 million, an accurate mass measurement could be performed. Accurate mass measurements could be made on the parent ion as well as fragments. The typical mass error was less than 5 ppm. Fragmentation spectra (MS/MS) were typically generated in a data-dependent manner, but if necessary, targeted MS/MS was employed, such as in the case of lower level signals.

Identification of known chemical entities was based on comparison to metabolomic library entries of purified standards. More than 1,000 commercially available purified standard compounds have been registered into a database for distribution to both the LC and GC platforms for determination of their analytical characteristics. The combination of chromatographic properties and mass spectra gave an indication of a match to the specific compound or an isobaric entity.

### Measurement of 2-hydroxyglutarate

Quantification of L/D-2-hydroxyglutarate (2-HG) in biological media/tissues was performed by LC-ESI-MS/MS as described [19] with modifications to accommodate different sample matrices involved in the study. The method utilizes a chiral derivatization agent to produce diastereoisomers with L- and D-isomers of 2-HG, which can be separated by conventional reverse-phase LC. D-2-HG, L-2-HG, and diacetyl-L-tartaric anhydride (DATAN) were from Sigma-Aldrich (St Louis, MO, USA). Racemic mixtures of L- and D-2-HG-d4 were prepared by mixing 1 mg of α-ketoglutarate-d6 (Sigma-Aldrich/Isotec) with 1 mg of NaBH_4_ (Sigma-Aldrich) in 0.2 mL anhydrous MeOH (Sigma-Aldrich) followed by 30 min incubation at 60°C. Tissue or cell line homogenates, 200 μL of deionized water, 1 mL of chloroform, and 4 mm ceramic beads were vigorously mixed for 45 sec at speed 4 in FastPrep 120 ‘bead-beater’ instrument (Thermo Savant, Holbrook, NY, USA). After centrifugation (5 min at 16,100 × g), 200 μL of the aqueous (upper) layer was transferred into 1.5-mL glass vial and dried (50°C, 60 min). The dry residue was treated with 50 mg/mL of freshly prepared DATAN in dichloromethane/glacial acetic acid (4/1 by volume) and heated at 75°C for 30 min. After drying (50°C, 15 min) the residue was dissolved in 100 μL LC mobile phase A (see below) for analysis by LC/MS/MS with an Agilent 1200 series HPLC (Agilent Technologies, St Clara, CA, USA) and Sciex/Applied Biosystems API 3200 QTrap (Applied Biosystems, Foster City, CA, USA). Mobile phase A: water, 3% acetonitrile, 280 μL ammonium hydroxide (approximately 25%), pH adjusted to 3.6 by formic acid (approximately 98%). Mobile phase B: methanol. Analytical column: Kinetex C_18_, 150 × 4.6 mm, 2.6 μm, and SafeGuard C_18_ 4 × 3 mm guard-column from Phenomenex (Torrance, CA, USA). Column temperature: 45°C. Elution gradient at 1 mL/min flow rate: 0 to 1 min 0% B, 1 to 2 min 0 to 100% B, 2 to 3.5 min 100% B, 3.5 to 4 min 100 to 0% B, 4 to 10 min 0% B. Injection volume: 10 μL. The Q1/Q3 (m/z) transitions monitored: 363/147 (2-HG) and 367/151 (2-HG-d4). A set of calibrator samples in corresponding matrix were prepared for calibration by adding appropriate amounts of pure D-2-HG at the following concentration levels: 0, 0.16, 0.54, 1.8, 6, and 20 ug/mL. These samples were analyzed alongside the experimental samples.

### Data analysis

For pairwise comparison of metabolites from different sample categories (normal, ER+, ER-), we used Welch’s *t* test. The false discovery rate was estimated using the *q* value [20].

The data were subjected to hierarchical clustering using Cluster 3.0 and displayed using TreeView [21]. The significance analysis of microarray (SAM) analyses were performed as described using indicated selection criteria [22]. For specific metabolite associations with genetic events, data were analyzed in GraphPad Prism (GraphPad Software, San Diego, CA, USA) for correlation and significance.

Genetic mutation and copy number, RNA expression data, and designation of tumor intrinsic subtype were all derived from the publically available TCGA data sets. Primary data were downloaded from the TCGA data portal [23] or analyzed using the online cBioPortal suite of tools [24]. For the cBioPortal, some analyses were performed on the ‘TCGA Nature 2012’ data set and others on the ‘TCGA Provisional’ data set.

For analysis of the correlation between each pair of metabolites, Pearson correlations of the level of each pair of metabolites (log2 normalized value) among 399 metabolites from 25 tumors and 5 normal breast tissues were generated. The correlation coefficients were hierarchically clustered by Cluster 3.0 to produce the heatmap plot. For analysis of correlation between individual metabolite and proliferation rate, Pearson correlations between the level of individual metabolite (log2 normalized value) and Ki-67% (log2 value) were calculated. The supervised cluster plot was generated based on the correlation between individual metabolite levels and proliferation rate (Ki-67%) and displayed by TreeView.

For combined analysis of receptor status and proliferation, normalized metabolic data were natural log transformed yielding a symmetric distribution of the data about the mean. We used normal theory linear regression to assess the extent to which the metabolic assay data could be predicted by one, the other or both of the tumor’s receptor status and proliferation rate (Ki-67%). We analyzed each metabolite separately and fit four models for each: (1) the model with intercept only; (2) the model with intercept and receptor status; (3) the model with intercept and log2 proliferation rate; and (4) the model with intercept, receptor status and log2 proliferation rate. We calculated analysis of variance tables for the two nested progressions of models: (1, 2, 4) and (1, 3, 4). We report the *P* values based on the associated F tests for (a) models 2 over 1, (b) 4 over 2, (c) 3 over 1 and (d) 4 over 3. The *P* values in (a) and (c) are for the regression of abundance of the metabolite on receptor status and for the regression of abundance of the metabolite on proliferation rate, respectively. The *P* values in (b) and (d) are for the regression of abundance of the metabolite on receptor status while adjusting for proliferation rate and for the regression of abundance of the metabolite on the proliferation rate while adjusting for receptor status, respectively.

## Results

To date, over 900 primary breast cancers have been profiled by the TCGA initiative over a four-year period using an evolving set of molecular analyses. For this reason, not all cancers were analyzed by all techniques. For the current metabolomic study, we chose 25 cancers that had passed quality control and were accepted for analysis by TCGA: 16 ER positive (all but two were also PR positive) and 9 cancers that were both ER and PR negative, determined by standard immunopathologic analysis after cytoreductive surgery. Of these, 23 cancers (15 ER + and 8 ER-) were eventually subjected to comprehensive genetic analysis by TCGA. In addition to the cancers, we selected 5 samples of normal breast tissue (from reduction mammoplasties not associated with cancer) that contained a substantial amount of epithelium based on histologic staining. We cut 20 sections from each block for *in situ* analyses before extraction for the quantitative profiling of small molecules (<1,000 Da) that was performed in a single batch on the Metabolon platform generating data on 399 identifiable metabolites (Table S2 in Additional file 2 contains the primary data on metabolites).

Primary tissues in this study were distributed into three main categories: (1) normal breast from reduction mammoplasties, (2) ER positive primary breast cancers, and (3) estrogen and PR negative primary cancers. Of the 25 cancers, all were accepted for TCGA study but only 23 were actually subjected to genetic analysis. The cancers are representative of the major subtypes of the disease based on the PAM50 classification [25] including basal, HER2, luminal A and luminal B. One sample was designated as ‘not-classified’ (NC). None of the tumors were classified as the relatively uncommon ‘claudin low’ subtype.

**Figure 1:**
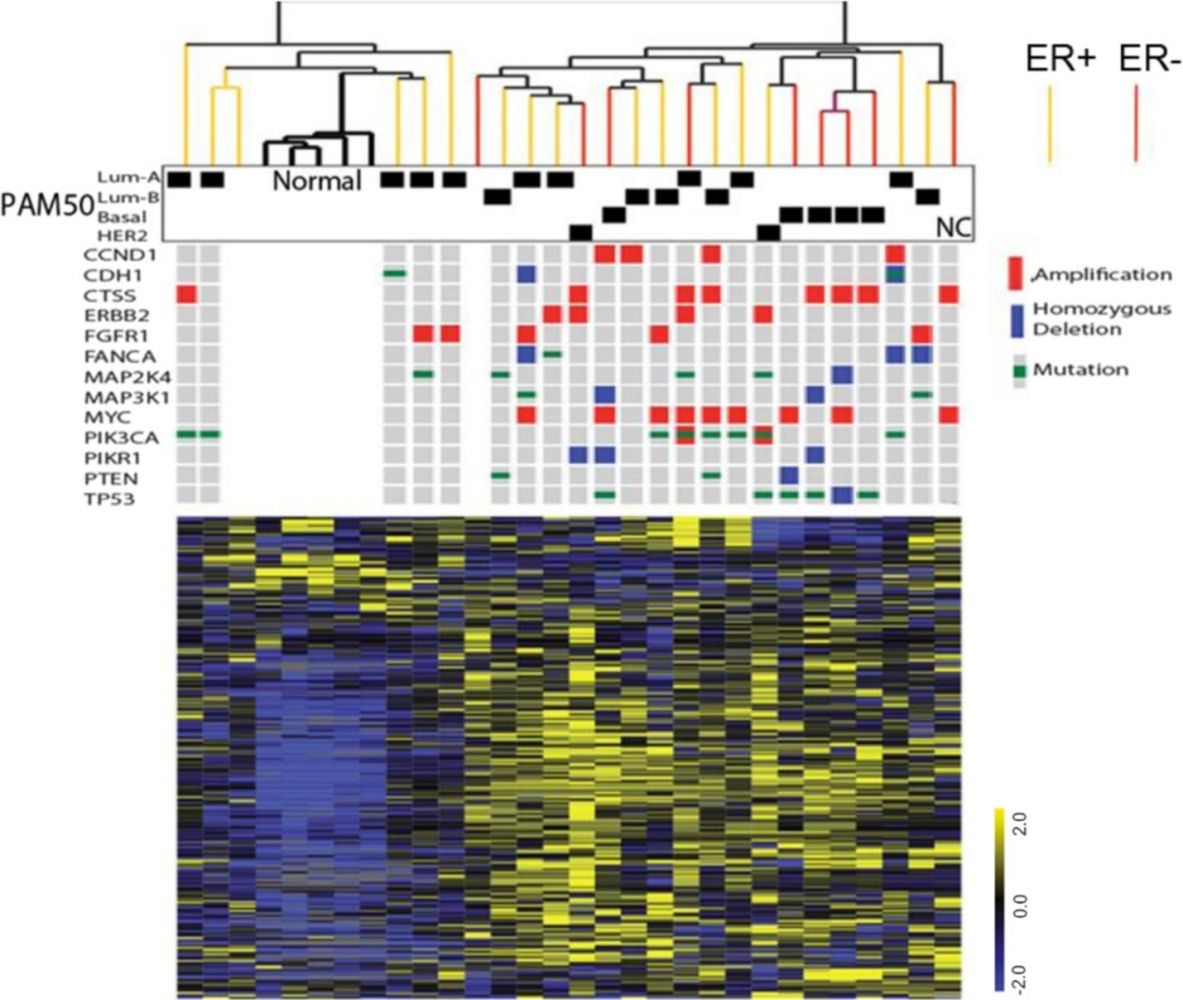
**Breast cancers (n = 25) and normal breast tissues (n = 5) were grouped by unsupervised hierarchical clustering of metabolite levels and overlaid with intrinsic subtype and status of the somatic mutations of genetic drivers.** Normalized metabolite levels were mean-centered, selected based on at least two-fold changes in two samples and arranged by hierarchical clustering. The estrogen receptor (ER) status, intrinsic subtypes, and identified somatic mutations in the indicated genes are shown for 23 tumors which were characterized by The Cancer Genome Atlas (TCGA).

Hierarchical clustering of the samples based on these metabolites demonstrated that the 5 normal breast samples were tightly clustered together while the ER + tumors exhibited significant heterogeneity: 6 of the ER + tumors grouped with the 5 normal breast samples while the remaining 10 ER + tumors clustered with the 9 ER- tumors (Figure 1). This relative heterogeneity of ER + tumors is consistent with previous classification based on gene expression [12],[26]. Pictured below the hierarchical clustering dendrogram for 23 of the cancers is TCGA data for the most common gene-based abnormalities (mutation, amplification, homozygous deletion) found in breast cancer, most of which are considered to be driver alterations. Also shown is the PAM50 intrinsic subtype classifier (based on gene expression) of the TCGA analyzed cancers (23 tumors) as well as the not-classified (NC) tumor. From this analysis, it is apparent that intrinsic expression tumor subtypes do not define the classification of the tumors by metabolite levels.

This composite diagram (Figure 1) demonstrates several aspects of breast cancer observed across many studies: (1) hormone receptor expression tracks closely with intrinsic subtype; (2) TP53 mutations are very frequent in basal-type cancers; and (3) PIK3CA mutations are common in luminal types. The metabolite-based clustering of these specimens places a group of luminal A cancers with the normal breast specimens and most of the basal cancers together in a single branch. A middle branch contains a combination of mostly luminal A and B cancers. HER2 cancers, as assigned by expression subtype (n = 2) or genomic copy number (n = 4) also tend to cluster in this middle branch. Overall, the cancers in the study appear to have a distribution of receptor status, intrinsic subtype, and genetic alterations typical of an unselected case series.

### Estrogen receptor status reflects a broad metabolic division in breast cancer

**Figure 2:**
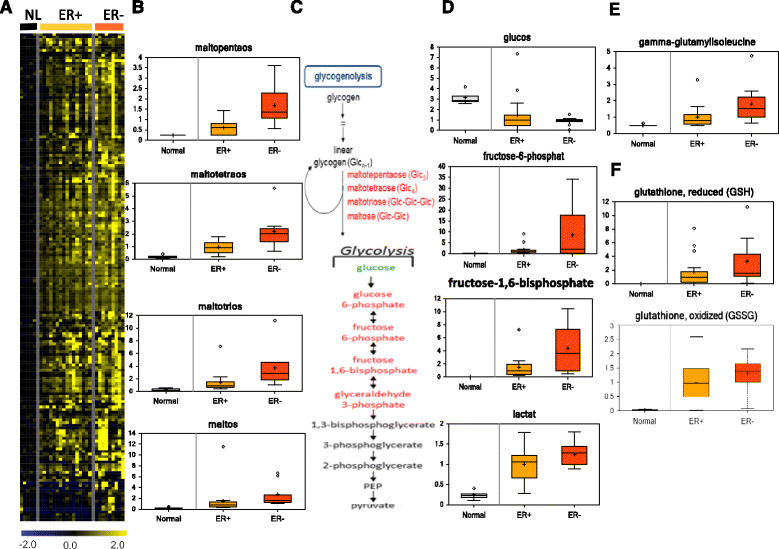
**Supervised analysis of metabolites by estrogen receptor (ER) status. (A)** Tumor-specific metabolites were zero-transformed against the mean of the five normal breast tumors, filtered and arranged by hierarchical clustering based on 16 ER + and 9 ER- tumors. **(B-D)** Significantly higher levels of metabolites in the glycogenolysis **(B)** and glycolysis **(D)** pathways, as shown in **(C)**, were found in the ER- compared to ER + tumors. The names of elevated (labeled in red) and reduced (labeled in green) metabolites in the glycolysis **(B)** and glycogenolysis **(D)** pathways are shown in the metabolism diagram **(C)**. Increased levels of gamma-glutamyl-isoleucine **(E)** and reduced (GSH) and oxidized (GSSG) glutathione **(F)** were also found. Primary data and *P* values for these comparisons can be found in Table S2 in Additional file 2 and Table S3 in Additional file 3.

Our next step in the analysis was to compare metabolite levels in the cancers based on ER status (ER + versus ER-), the most consistent division in breast cancer from both a biologic and therapeutic perspective (Figure 2A). Overall, ER status was a very strong divisor in metabolic space. The identity of the metabolites that vary by ER status supports a series of systematic differences in bioenergetics and biosynthetic pathways. Of the 399 named metabolites quantified in this study, 75 exhibited a statistically significant difference between ER + and ER- tumors (Table S3 in Additional file 3, unadjusted *t* tests comparing levels between the three groups of samples, ER+, ER-, and normal breast). Of these, only 8 metabolites were increased in ER + tumors including 3 carnitine derivatives, suggesting an increase in fatty acid transportation in hormone receptor positive cancers. Short- and medium-chain fatty acids were also elevated in ER + tumors whereas long-chain fatty acids and monoacylglycerols tended to be higher in ER- tumors indicating that systematic differences in lipolysis and fatty acid oxidation correlate with hormone receptor status.

ER- tumors had higher levels of glycogenolytic (maltopentose, maltotetraose, maltotriose and maltose, Figure 2B and pathways in Figure 2C) and glycolytic metabolites (glucose-6-phosphate, fructose-6-phosphate, fructose-1,6-bisphosphate, and lactate) (Figure 2D). In contrast, there was a lower level of glucose in the tumors, especially ER- tumors (Figure 2D). Warburg metabolism is a means for rapidly dividing cells, such as cancer cells, to accelerate energy production through increased glycolysis and lactate production, bypassing the normal oxidation of pyruvate in the mitochondria. Thus, the metabolic profile of ER- tumors is consistent with an elevated Warburg effect.

To validate our results, we compared our data to recently published data on breast cancers collected using the same metabolomic platform [27]. Among the significant metabolites that tracked with hormone receptor status in our data, 57 of these were found in the Terunuma dataset and exhibited broadly similar differences between ER + and ER- cancers (27 reaching significance in both data sets, Figure S1 in Additional file 4 and Table S4 in Additional file 5). Therefore, these identified subtype-specific metabolites can be validated using an independent dataset.

### Reduced glutathione (GSH) and gamma-glutamyl amino acids in ER- tumors

ER- cancers also had higher levels of gamma-glutamyl amino acids coupled with increased glutathione synthesis (Figure 2E, Figure S2 in Additional file 4). Gamma-glutamyl amino acids result from the transpeptidase-mediated catalytic reaction of amino acids with glutathione (Figure S2 in Additional file 4). These amino acid-glutathione conjugates traverse the cell membrane and release the amino acid intracellularly to regenerate glutathione [28]. Elevated levels of these gamma-glutamyl conjugates indicate an increased uptake of amino acids in ER- tumors. This may point to a potential shift in fuel substrates for energy production that favors amino acid catabolism. It is interesting to note that the most elevated gamma-glutamyl amino acids were the branched-chain amino acid (BCAA) conjugates of valine, leucine and isoleucine. Additionally, ER- tumors had increased glutathione (reduced, GSH) and oxidized glutathione (GSSG) (Figure 2F) indicating a trend toward increased glutathione synthesis, presumably to cope with the higher levels of oxidative stress.

### Metabolite correlations in breast cancers

**Figure 3:**
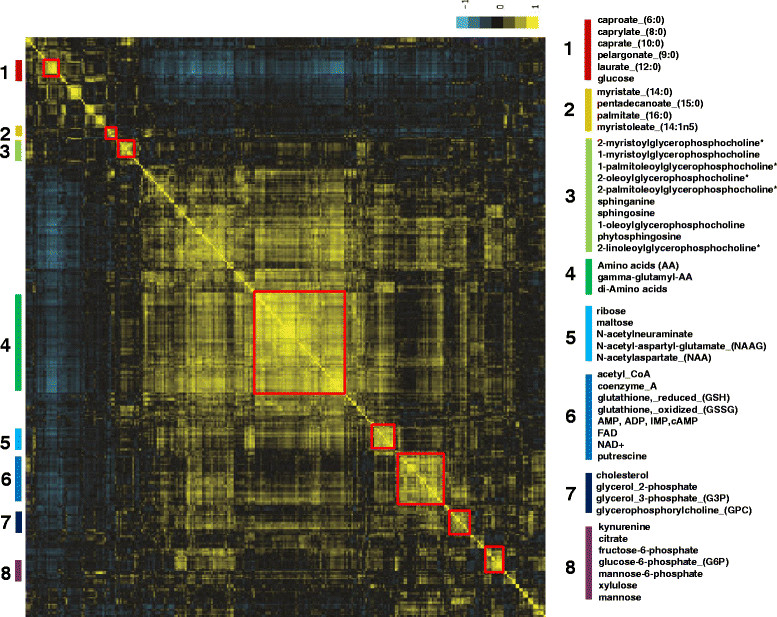
**Hierarchical clustering of metabolites based on correlation coefficients.** The correlation coefficients were calculated using Pearson product-moment of each pair of metabolites (log base 2 normalized) among 399 metabolites from 25 breast cancers and 5 normal breast tissues. Eight clusters of highly correlated metabolites are highlighted on the right panel.

We postulated that the level of multiple metabolites derived from the same and different metabolic pathway might be coordinated and serve as a better indicator of metabolic activity than any single compound alone. Pearson correlations were calculated for all pairwise comparisons between each of the 399 metabolites for all samples (Table S5 in Additional file 6). The resulting correlation coefficients were then used to group the 399 metabolites into distinct groups by hierarchical clustering (cluster 3.0) and then displayed with TreeView (Figure 3). We found that multiple groups of metabolites were highly clustered and correlated. These groups include many metabolites that are known to be in the same metabolic pathways as well as unexpected correlation between metabolites in different metabolic pathways. Metabolites from different pathways clustered in the same groups might indicate two different metabolic pathways are coordinated by the same genetic alteration or affected similarly by the metabolic reprogramming. For example, we found a cluster of metabolites comprising many intermediates of various lipids associated with glycerophosphocholines (Figure 3, cluster 3). We also noted two separate clusters of amino acids and di-amino acids (glycine-proline, glutamate-leucine, alanine-tyrosine) (Figure 3, cluster 4) and N-acetyl-amino acids (N-acetyl-aspartate, N-acetyl-ornithine, N-acetyl-aspartyl-glutamate) (Figure 3, cluster 5). Both clusters may indicate products of protein degradation and catabolism and can be used to identify tumors with higher protein catabolism.

Another prominent cluster is composed of acetyl-CoA, CoA, FAD, AMP as well as GSH and GSSH (Figure 3, cluster 6). The co-cluster of these metabolites suggests a high degree of correlation between these energy metabolites and anti-oxidative capacity among the tumor tissues. We also noted connected clusters of metabolites related to glycolysis (fructose, glucose-6-phosphate, fructose-6-phosphate) and glycogenolysis (malto-triose, malto-tetraose, malto-pentose) (Figure 3, cluster 8), both were elevated in ER- cancers (Figure 2).

### Metabolites associated with specific genetic events

**Figure 4:**
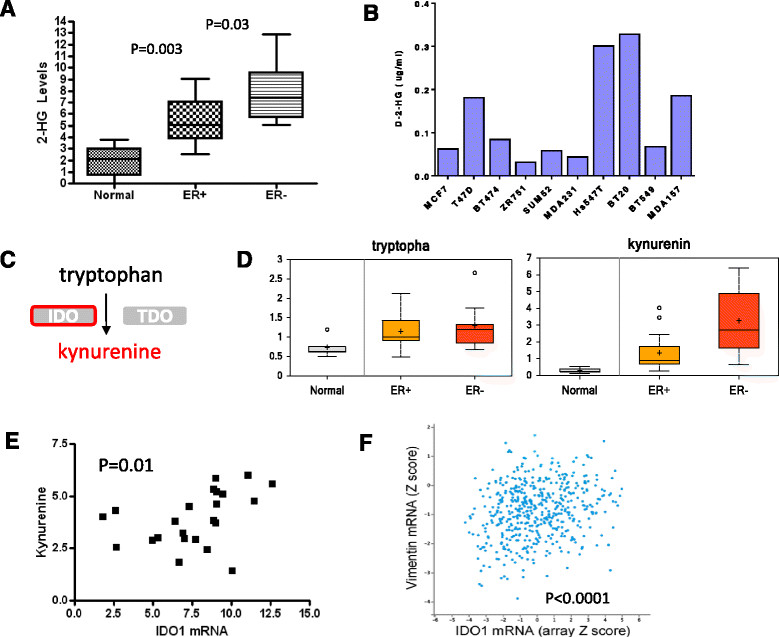
**Specific metabolic/genetic associations. (A)** The mean level of 2-hydroxyglutarate (2-HG) in 5 normal tissues, 16 estrogen receptor (ER) + and 9 ER- tumors. **(B)** The level of 2-HG in breast cancer cell lines (4 luminal type and 5 basal type cells). **(C)** Kynurenine is derived from tryptophan by indoleamine 2,3-dioxygenase (IDO) or tryptophan 2,3-dioxygenase (TDO) enzymatic activity. **(D)** The level of tryptophan and kynurenine in 5 normal tissues, 16 ER + and 9 ER- tumors. **(E)** The correlation between the level of kynurenine and IDO1 gene expression measured by RNAseq in 23 tumors analyzed by The Cancer Genome Atlas (TCGA). **(F)** The correlation between RNA expression of the mesenchymal/basal marker vimentin and IDO1 in TCGA breast cancers.

A novel and unexpected finding was the level of the oncometabolite 2-HG, elevated over 20-fold in ER + tumors compared with normal tissue and over 200-fold in ER- tumors (Figure 4A). A single ER- tumor exhibited 10-fold higher levels of 2-HG compared to any other sample (unscaled data). We further confirmed the levels of 2-HG in a subset of the breast cancer extracts using an independent assay based on MS performed at Duke and found a very high correlation between the results from these two independent platforms (Figure S3A in Additional file 4). Using this targeted assay, we also measured 2-HG in a series of breast cancer cell lines and found that two basal lines (Hs578T and BT20) had the highest levels, consistent with data from the primary cancers (Figure 4B). A high level of 2-HG has been associated with missense mutations in IDH1 or IDH2 in glioma and several other tumor types [29] and may be an effector of tumor cell dedifferentiation [30]. Within the TCGA breast data set, two cancers (<0.5%) were found to harbor missense mutations in IDH1 that have a high probability of affecting the function of the enzyme (R132C, Y235C). Since these data were based on whole genome sequencing that could overlook specific mutations, we performed targeted sequencing in the tumor (TCGA-B6-A1KF) with very high levels of 2-HG to look for the recurrent mutations found in other tumors in IDH1 and IDH2. This tumor was wild type for both genes in these regions (Figure S3B in Additional file 4) suggesting an alternative mechanism leading to 2-HG production, such as the recently reported activation of the myc pathway [27]. Levels of IDH1 and IDH2 mRNA in these specimens also did not show a significant correlation with 2-HG levels.

A number of reports indicate that alterations in specific genes or genetic pathways can result in detectable metabolic changes in cancer. Our study with both detailed gene expression and metabolomic data provided a powerful means to test these associations and discover new ones. One of the least complex of such relationships is the link between the level of the tryptophan-degrading enzyme indoleamine 2,3-dioxygenase (IDO1) and levels of the immunomodulatory metabolite, kynurenine (Figure 4C). Whereas the precursor molecule tryptophan did not vary between ER + and ER- cancers, median levels of kynurenine were significantly elevated in ER- tumors (Figure 4D). From RNAseq data of the 23 cancers in our study, we found a significant correlation between IDO1 expression and kynurenine levels (Figure 3E, r = 0.55, *P* = 0.01). Comparing levels of IDO1 mRNA within the TCGA data set (n = 748) revealed a significant positive correlation of IDO1 mRNA levels with vimentin expression (a hallmark of basal cancers). This finding suggests that kynurenine accumulation commonly occurs in basal cancers and could lead to reduced immunosurveillance in this tumor type.

**Figure 5:**
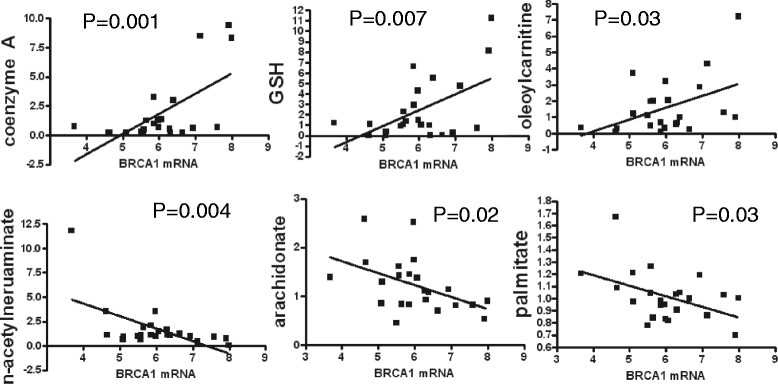
**Correlation between selected metabolite levels and BRCA1 mRNA expression.** Three representative metabolites (coenzyme A (CoA), reduced glutathione (GSH) and oleoyl-carnitine) that are positively and three (N-acetylneuraminate, arachidonate and palmitate) that are inversely correlated with BRCA1 mRNA levels from The Cancer Genome Atlas (TCGA) RNAseq data on 23 cancers are shown. The full list of metabolites correlated with BRCA1 is provided in Table S6 in Additional file 7 showing listing both normalized metabolite levels and metabolite levels with an additional log2 transformation to reduce the impact of outliers.

BRCA1 has been implicated in a number of metabolic processes including fatty acid synthesis and response to oxidative stress. Using the available RNAseq data, we correlated expression of BRCA1 with metabolite levels in the 23 TCGA cancers (Table S6 in Additional file 7). There was evidence of strong association between high levels of BRCA1 mRNA and elevated acetyl CoA, CoA, 3′ dephospho-CoA, and several acylcarnitines all indicative of higher levels of fatty acid β-oxidation (Figure 5). This is consistent with the reported ability of BRCA1 to inhibit acetyl-coenzyme A carboxylase 1 (ACC1/ACACA) leading to reduced fatty acid synthesis and increased fatty acid β-oxidation resulting in the accumulation of acetyl-CoA and CoA [31]. In contrast, there were strong inverse correlations between BRCA1 levels and membrane components, long-chain fatty acids, and amino acids further supporting the role of BRCA1 in regulating the balance between fatty acid synthesis and oxidation (Figure 5) [32]. In addition, GSH and another antioxidant, 3-(4-hydroxyphenyl)lactate were also positively correlated with BRCA1 mRNA levels, supporting its role in activating NRF2, the master regulator of the oxidative stress response and GSH synthesis [33]-[35]. BRCA1 mRNA levels did not correlate with proliferation or ER status indicating that this is an independent set of variables. Furthermore, the BRCA1 mRNA did not correlate with the mRNA levels of genes in the fatty acid biosynthesis pathways; consistent with posttranscriptional regulation (Figure S4 in Additional file 4).

To further explore associations with the most common genetic events in breast cancer, we performed SAM analyses (Table S7 in Additional file 8) to identify metabolites associated with the somatic driver alterations (shown in Figure 1, used as categorical variables that is, mutant versus wild type, amplified/deleted versus diploid). Many of the genetic events are loosely associated with specific tumor subtypes (for example, PIK3CA mutations were not found in basal cancers) such that metabolite correlations with specific genetic features may be confounded by higher level associations. With this in mind, the results do support several relationships between metabolite profiles and tumor genetics. Most notably, compared to tumors with wild-type p53, cancers with TP53 alterations show a very specific pattern of decreased lipid glycerophosphocholines that is not apparent when classifying the cancers by ER status, (Figure S5 in Additional file 4). Other significant associations were observed between PIK3CA mutation and malonylcarnitine and ERBB2 amplification with docosapentaenoate, fucose, and 1-oleoylglycerophosphoethanolamine (Figure S6 in Additional file 4).

### Metabolites associated with proliferation in tumors

**Figure 6:**
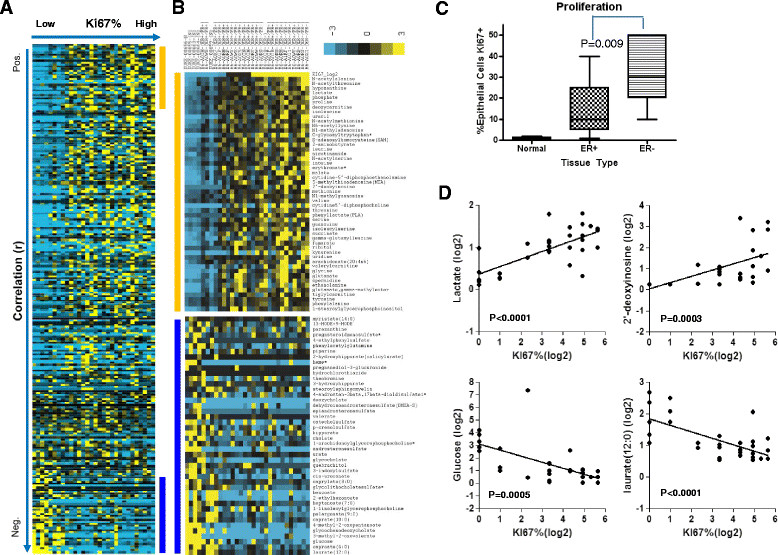
**Proliferation rate is correlated with the level of many metabolites. (A)** Supervised clustering of normalized metabolite levels as ordered by the proliferation rate (Ki-67%) (from low (left) to high (right)). **(B)** Zoomed views of the metabolites that are most positively (orange bar) or negatively (blue bar) correlated with proliferation. **(C)** Proliferation associated with receptor status and cancer status from the 30 samples in this study. **(D)** The correlation of representative metabolites positively or negatively associated with proliferation rate.

Proliferation rate could impact the level of many metabolites. We measured proliferation by *in situ* detection of Ki-67 followed by quantitative evaluation of the percentage of epithelial cells positive for this antigen. As previously demonstrated in many studies, proliferation tends to be significantly higher in ER- compared to ER + tumors (mean of 32% versus 14%, *P* = 0.009 in our cohort) and epithelial cells in normal breast tissue have a very low rate of proliferation (Figure 6C). Supervised clustering and correlation analysis of the metabolites with proliferation rate demonstrated sets of metabolites that were positively and inversely correlated with proliferation (Figure 6A and B, and Table S8 in Additional file 9). Predictably, the level of glucose was lower in rapidly proliferating cancers whereas lactate was positively correlated with proliferation (Figure 6D). N-acetyl amino acids and 2′-deoxyinosine were highly enriched in rapidly proliferating tumors. The biological roles of N-acetyl amino acids are largely unknown. However, aminoacylase-1 (encoded by ACY1), which is responsible for the degradation of these N-acetyl amino acids, has been found to be inactivated in several tumor types [36],[37]. High levels of 2′-deoxyinosine (dI) may be an indication of misincorporation of dI into the DNA of ER- tumor cells, a lesion capable of generating A-- > G transitions in DNA [38].

ER status was highly correlated with proliferation and therefore many of the same metabolites were associated with both of these parameters. We further analyzed the data to determine whether these two parameters had any degree of independence, and if so, for which sets of metabolites. Linear regression analyses show that, while correlated with one another, ER and proliferation status act as complementary explanatory factors for many of the metabolites (Figure S7 in Additional file 4). A relatively small number of metabolites were highly correlated with one parameter but not the other, most notably 2-HG with ER status but not proliferation (Table S9 in Additional file 10).

## Discussion

Links between cancer genetics and altered metabolism have been established primarily in model and experimental systems but have rarely been tested in primary human cancers. The current study makes use of the comprehensive genetic data from TCGA on breast cancer [1] to test reported relationships and discover new and unexpected associations between genetics and metabolism. TCGA data provides an excellent platform in this regard for three reasons: (1) the tissues were subjected to stringent quality control criteria including tumor nuclei exceeding 70% and the absence of significant necrosis, (2) multiple analytes were measured in parallel on the same cancers including DNA sequence for mutations, DNA copy number assessment, RNA expression including RNAseq and microarray analysis, methylation, and protein and phosphoprotein levels, and (3) the data are in the public domain in easily accessible formats with standardized specimen identifiers that can be directly linked and co-analyzed with other types of newly generated data such as the metabolite levels that we measured in the current study. We include the primary data on these samples so that anyone can perform their own joint analysis on metabolites and genes of interest.

The frozen tissues were analyzed on a metabolomics platform that has been used in other cancer-related studies [39]-[41] and at the time of the analysis, included identification and quantitation of 399 named biochemicals (M_r_ <1,000 Da). Unsupervised clustering by metabolite levels revealed two major categories, one containing the normal breast tissues and a subset of the ER + cancers, and the other containing all of the ER- cancers and the remaining ER + ones. Overlaying the TCGA data on these clusters revealed that all of the cancers clustering with normal breast were of the luminal A intrinsic subtype. The remaining luminal A and all of the luminal B cancers fell in the other major cluster along with all of the basal cancers and the two cancers designated as HER2 by PAM50. That the luminal A cancers do not all cluster together is consistent with a metabolomic study employing high resolution magic angle spinning magnetic resonance spectroscopy of predominantly luminal cancers [17]. In this study, three distinct categories of luminal A cancers were described by hierarchical clustering driven primarily by varying levels of glycolytic activity. Widely different proliferation rates in the ER + cancers may at least partially underlie these luminal sub-clusters.

The basal subtype, a subset of the ER- cancers, demonstrated significant homogeneity with 4 of the 5 PAM50 basal cancers clustering on one sub-branch of the metabolomic hierarchical tree. A HER2 cancer was the only other member of this sub-branch but this cancer does share the common basal trait of having a TP53 mutation. Overlaying the most common genetic driver alterations in breast cancer on this cluster diagram allowed a visual assessment of whether branches may also be driven by specific oncogenic events. TP53 mutations and basal cancer status are nearly coincident and as described, the basal cancers are tightly clustered. Therefore, it is difficult to disentangle genetics from intrinsic subtype in this instance. Other notable groupings that may be associated with a specific genetic driver include MYC amplification and PIK3CA mutation. PIK3CA mutations are tightly linked to the luminal subtypes whereas MYC amplification is common in both basal and luminal cancers. It may be noteworthy that none of the MYC amplified tumors clustered with the normal breast tissue.

Supervised classification and *t* tests based on the three categories of specimens (ER+, ER/PR-, normal) exhibited significant signals for many metabolites. Over half of the 399 identified metabolites were different between normal and cancer. Moreover, >18% of the metabolites varied significantly between ER + and ER- cancers. These dramatic differences are consistent with previous reports showing strong remodeling of central metabolism between normal breast tissue and breast cancer [16] and between tumor subtypes [9]. Notably, glycolytic- and glycogenolytic-associated metabolites including lactate were higher in ER- cancers with the prominent exception of low tumor glucose. The increased lactate production from ER- cancer may be caused by increased glycolysis and confirm our previous finding of a strong hypoxia program and the high ‘Warburg’ phenotype previously described in other studies of ER- cancer [7],[9],[16],[27].

From TCGA data (for 23 of 25 of the cancers), we were able to confirm or identify known and unexpected associations between metabolite levels and various genetic events. Proof of principle for this approach was demonstrated by the correlation between kynurenine and the mRNA of its synthetic enzyme, IDO1. IDO1 expression was also found to correlate with the basal phenotype common to ER- cancers and the high levels of kynurenine produced could result in reduced immunosurveillance of these cancers [42]. Increased serum kynurenine/tryptophan ratio has been noted during the progression of several tumor types [43],[44]. Our findings support a connection between high IDO levels and kynurenine in ER- tumors. Other *a priori* associations that were tested included PKM2 exon 9 versus 10 splice variant levels with proliferation and metabolite levels [45], IDH1 and 2 mRNA and mutations with 2-hydroxyglutarate levels [29], and levels of GGT1 mRNA and the gamma-glutamyl amino acids [46]. Of these, the strongest association was between GGT1 and a subset of the conjugated amino acids including leucine and isoleucine (Figure S8 in Additional file 10). GGT1 expression has been associated with a subset of basal cancers and our data indicating elevated levels of gamma-glutamyl amino acids in ER- cancers provides functional support for this genetic link.

A number of driver mutational events have been implicated in breast cancer development including amplification of ERBB2, CCND1 and MYC, loss of PTEN and CDH1, and missense mutations in TP53 and PIK3CA. Some of these events have been associated with changes in central metabolism in tumors and experimental systems [4],[5],[47]-[49]. Using these genetic alterations as categorical variables, we analyzed our data for signs of metabolic signatures associated with the most common genetic lesions. The co-existence of TP53 mutation and reduced levels of a series of lipid glycerophosphocholines was the most significant association detected. Altered choline metabolism in the form of decreased glycerophosphocholine (GPC) was reported for several tumor types, consistent with an association with p53 status [50],[51]. A specific connection between p53 and phospholipid metabolism was demonstrated previously with indication of feedback regulation between phospholipid turnover and p53 activity [52]-[55]. In our data, 8 different long-chain fatty acid glycerophosphocholines were strongly reduced in p53 mutant tumors suggesting an underlying regulatory relationship for this consistent association. An important caveat for this finding is the fact that p53 mutation status is associated with the basal intrinsic subtype in breast cancer. In our data set, all five of the basal cancers harbored p53 mutations and only one of the nonbasal cancers (HER2) had a mutation. Therefore, the strong association we observed between TP53 status and the levels of these phospholipids may be confounded by intrinsic subtype something that cannot be distinguished in the current study. Another interesting metabolite-genetic association is the higher level of fucose in ERBB2+ tumors. Fucose is a simple sugar that is used to modify proteins and shown to be necessary for key functions of neoplastic progression of breast cancer cells [56]. The higher level of fucose may suggest that such glycoprotein modifications play a particular important role in ERBB2+ tumors.

Our findings with respect to BRCA1 further highlight the potential of these joint analyses. Germ line BRCA1 mutations typically lead to triple-negative cancers, but broad variation in mRNA expression is also observed outside of the context of the hereditary syndrome. High levels of BRCA1 mRNA were positively correlated with a group of metabolites indicative of elevated fatty acid β-oxidation and increased anti-stress response and inversely correlated with medium- and long-chain fatty acids and membrane components. BRCA1 protein via its BRCT domain was shown to bind to ACC1/ACACA preventing its dephosphorylation, keeping it in a phosphorylated and inactive form, thus inhibiting fatty acid synthesis and promoting fatty acid β-oxidation [31]. We found a number of metabolites that fit this mechanism. BRCA1 has also been implicated in redox homeostasis [33], potentially through an interaction with NRF2 that prevents its degradation and promotes its nuclear accumulation [35] and we found multiple metabolites that also fit this activity. These BRCA1 expression-metabolite associations are entirely consistent with *in vitro* mechanistic studies implicating BRCA1 in these processes and as such, constitute direct support for the physiologic relevance of these pathway connections in breast cancer.

Rate of proliferation is a key phenotypic property of breast cancers that can be used as an independent prognostic factor [57]. Gene expression indicative of proliferation constitutes a major component of the OncotypeDx multi-gene recurrence score [58]. We measured proliferation in our specimens by scoring Ki-67 staining as a continuous variable and correlated this metric with metabolite levels. As anticipated, a number of strong correlations with proliferative rate were found including high levels of lactate and low levels of glucose consistent with glucose-consumption patterns in rapidly growing cells. Overall, many of the same metabolites correlated with both receptor status and proliferation. While proliferation did correlate with ER status in our study (and others), there were ER + cancers with high proliferation rates and ER- cancers with relatively low proliferation rates in our sample set. We compared the relative contribution of these two factors (proliferation and receptor status) to metabolite levels through statistical analysis and found that a number of analytes were strongly associated with one parameter but not the other suggesting that there is some degree of independence. However, these results further highlight the potential confounding classification issue in breast cancer as receptor status, intrinsic subtype, and genetic and phenotypic properties are all correlated. The admixture of cancers we analyzed in the current study reinforces these relationships indicating that this data set is highly representative of the landscape of the disease. The inclusion of metabolite profiles as an additional dimension in the TCGA database provides a new level of resolution to this important public resource. Indexing our metabolite data to the rigorously curated, comprehensive, and standardized TCGA platform offers the opportunity for additional hypothesis testing and discovery based upon metabolic signatures and could produce novel insights for detection, prognostic, predictive, or therapeutic benefit.

## Conclusions

We have identified categorical differences in the metabolic profile of ER- vs. ER + breast tumors that may directly impact tumor behavior and clinical phenotypes. We found notable differences in energy needs, redox potential, protein uptake and catabolism which in the ER- samples correlated with increases in glutathione biosynthesis, NAD + production, and proliferative signaling. The data are consistent with high Warburg metabolism in the ER- tumors, as several biochemical intermediates of the glycolytic pathway including lactate were found to be increased in these cancers. Joint analysis with genetic alterations further identified several gene-metabolite correlations validating the physiologic relevance of reported *in vitro* associations and providing indications of novel regulatory relationships between tumor genetics and metabolism. The addition of metabolomic data to the public domain TCGA dataset provides an important new tool for the discovery and hypothesis testing of the genetic regulatory of tumor metabolism.

## Additional files

## Electronic supplementary material

Additional file 1: Table S1.: Demographic and clinical data for the breast cancer subjects from which the tissues for this study were derived. (DOCX 15 KB)

Additional file 2: Table S2.: Metabolite levels (normalized and imputed) of the breast samples with genetic driver alterations indicated. (XLSX 144 KB)

Additional file 3: Table S3.: *t* tests comparing metabolite levels between the three groups of specimens; normal breast, estrogen receptor (ER) + cancers, and ER- cancers. (XLSX 67 KB)

Additional file 4: Figure S1.: Metabolomic comparison between our dataset and Terunuma *et al*.’s dataset. Lipid-containing metabolites are highlighted in blue typeface. **Figure S2.** Significantly higher levels of several metabolites in the cysteine and glutathione homeostasis and amino acid cycle were found in estrogen receptor (ER)- compared to ER + tumors. **Figure S3. (A)** Highly correlated 2-hydroxyglutarate (2-HG) level measured by two different laboratories and methods. On the y-axis are the values from Metabolon and the x-axis are the same samples analyzed in the Duke Cancer Pharmacology Laboratory. The right hand plot normalizes the scales for each set of measurements to spread out the points. **(B)** Absence of IDH1 and IDH2 mutations in very high 2-HG sample (TCGA-B6-A1KF). For comparison, the IDH1 R132C mutation is shown from HT1080 cells. **Figure S4.** Heatmap of expression of BRCA1 and genes in fatty acid biosynthesis pathway based on The Cancer Genome Atlas (TCGA) mRNA data. **Figure S5.** A series of lipid glycerophosphocholines significantly reduced in cancers with TP53 mutations. **Figure S6.** Examples of metabolites significantly higher in tumors with mutant PIK3CA or ERBB2 amplified tumors. **Figure S7.** Scatter plots of the associations of each metabolite with log proliferation versus the same for receptor status given that log proliferation status is already accounted for in the model (and vice versa). Each point corresponds to a metabolite and the color corresponds to the overall strength of association between receptor and proliferation and the metabolite. The dashed red lines correspond to *P* = 0.05. Note that two of the four metabolites (in each analysis) most highly associated with proliferation or receptor status show significant additional explanatory ability for receptor status (red dots); these metabolites represent the strongest overall associations. **Figure S8.** Correlation between GGT1 mRNA levels and selected gamma-glutamyl amino acids. (PPTX 7 MB)

Additional file 5: Table S4.: Comparison with the Terunuma *et al*.’s (JCI 2014) dataset for discrimination of estrogen receptor (ER) + and ER- cancers. (XLSX 16 KB)

Additional file 6: Table S5.: Pearson’s correlations between all metabolites in the study. (XLSX 865 KB)

Additional file 7: Table S6.: Pearson’s correlations between BRCA1 mRNA levels and metabolites for 23 cancers comparing normalized metabolite levels and then log2 transformed normalized metabolite levels with RNAseq expression data from The Cancer Genome Atlas (TCGA). The metabolites that are significantly associated with BRCA1 mRNA levels are labeled in red (positive correlation) or green (negative correlation). (XLSX 50 KB)

Additional file 8: Table S7.: Significance of microarray (SAM) analysis of metabolites using each genetic driver alteration as a separate binary condition. (XLSX 418 KB)

Additional file 9: Table S8.: Pearson’s correlations between KI-67 (proliferation rate) and metabolite levels. (XLSX 27 KB)

Additional file 10: Table S9.: Linear regression analyses of proliferation (KI-67), estrogen receptor (ER) status, and metabolite levels. (XLSX 65 KB)

Below are the links to the authors’ original submitted files for images.

Authors’ original file for figure 1

Authors’ original file for figure 2

Authors’ original file for figure 3

Authors’ original file for figure 4

Authors’ original file for figure 5

Authors’ original file for figure 6

## Abbreviations

2-HG: 2-hydroxyglutarate
ACC1: acetyl-coenzyme A carboxylase 1, encoded by ACACA (acetyl-CoA carboxylase alpha)
ER: estrogen receptor
GC/MS: gas chromatography/mass spectrometry
GSH: glutathione, reduced
GSSG: glutathione, oxidized
HER2: amplified ERBB2 oncogene
IDO: indoleamine 2,3-dioxygenase
LC/MS: liquid chromatography/mass spectrometry
NMR: nuclear magnetic resonance
NFE2L2: NFE2-related factor 2
PAM50: 50 gene expression signature separating breast cancers into 5 intrinsic subtypes
PR: progesterone receptor
TCGA: The Cancer Genome Atlas
