# Supplementary material for: A joint analysis of metabolomics and genetics of breast cancer
Source: Breast Cancer Res. 2014 Aug 5;16:415. doi: 10.1186/s13058-014-0415-9 (PMC4187326; doi:10.1186/s13058-014-0415-9)
Supplement: Supplementary file 1 — Additional file 1: Table S1.: Demographic and clinical data for the breast cancer subjects from which the tissues for this study were derived. (DOCX 15 KB) [file 13058_2014_415_MOESM1_ESM.docx]

|  | ER+ | ER- |
| --- | --- | --- |
| Age (median) | 61 | 40 |
| Race |  |  |
| Caucasian | 10 | 7 |
| AA | 6 | 2 |
| Tumor Size |  |  |
| ≤2cm | 4 | 0 |
| >2cm | 10 | 9 |
| T_X_ | 2 | 0 |
| Nodal Status |  |  |
| Negative | 4 | 3 |
| Positive | 8 | 6 |
| N_X_ | 4 | 0 |
| Median Follow-up | 102 months | 89 months |
| Chemotherapy |  |  |
| Yes | 4 | 7 |
| No | 12 | 2 |
| Hormonal Therapy |  |  |
| Yes | 11 | 1 |
| No | 5 | 8 |
|  |  |  |

Supplemental Table 1. Clinical and demographic characteristics.
