# Supplementary material for: A joint analysis of metabolomics and genetics of breast cancer
Source: Breast Cancer Res. 2014 Aug 5;16:415. doi: 10.1186/s13058-014-0415-9 (PMC4187326; doi:10.1186/s13058-014-0415-9)
Supplement: Supplementary file 4 — Additional file 4: Figure S1.: Metabolomic comparison between our dataset and Terunuma et al.’s dataset. Lipid-containing metabolites are highlighted in blue typeface. Figure S2. Significantly higher levels of several metabolites in the cysteine and glutathione homeostasis and amino acid cycle were found in estrogen receptor (ER)- compared to ER + tumors. Figure S3. (A) Highly correlated 2-hydroxyglutarate (2-HG) level measured by two different laboratories and methods. On the y-axis are the values from Metabolon and the x-axis are the same samples analyzed in the Duke Cancer Pharmacology Laboratory. The right hand plot normalizes the scales for each set of measurements to spread out the points. (B) Absence of IDH1 and IDH2 mutations in very high 2-HG sample (TCGA-B6-A1KF). For comparison, the IDH1 R132C mutation is shown from HT1080 cells. Figure S4. Heatmap of expression of BRCA1 and genes in fatty acid biosynthesis pathway based on The Cancer Genome Atlas (TCGA) mRNA data. Figure S5. A series of lipid glycerophosphocholines significantly reduced in cancers with TP53 mutations. Figure S6. Examples of metabolites significantly higher in tumors with mutant PIK3CA or ERBB2 amplified tumors. Figure S7. Scatter plots of the associations of each metabolite with log proliferation versus the same for receptor status given that log proliferation status is already accounted for in the model (and vice versa). Each point corresponds to a metabolite and the color corresponds to the overall strength of association between receptor and proliferation and the metabolite. The dashed red lines correspond to P = 0.05. Note that two of the four metabolites (in each analysis) most highly associated with proliferation or receptor status show significant additional explanatory ability for receptor status (red dots); these metabolites represent the strongest overall associations. Figure S8. Correlation between GGT1 mRNA levels and selected gamma-glutamyl amino acids. (PPTX 7 MB) [file 13058_2014_415_MOESM4_ESM.pptx]

## Slide 1
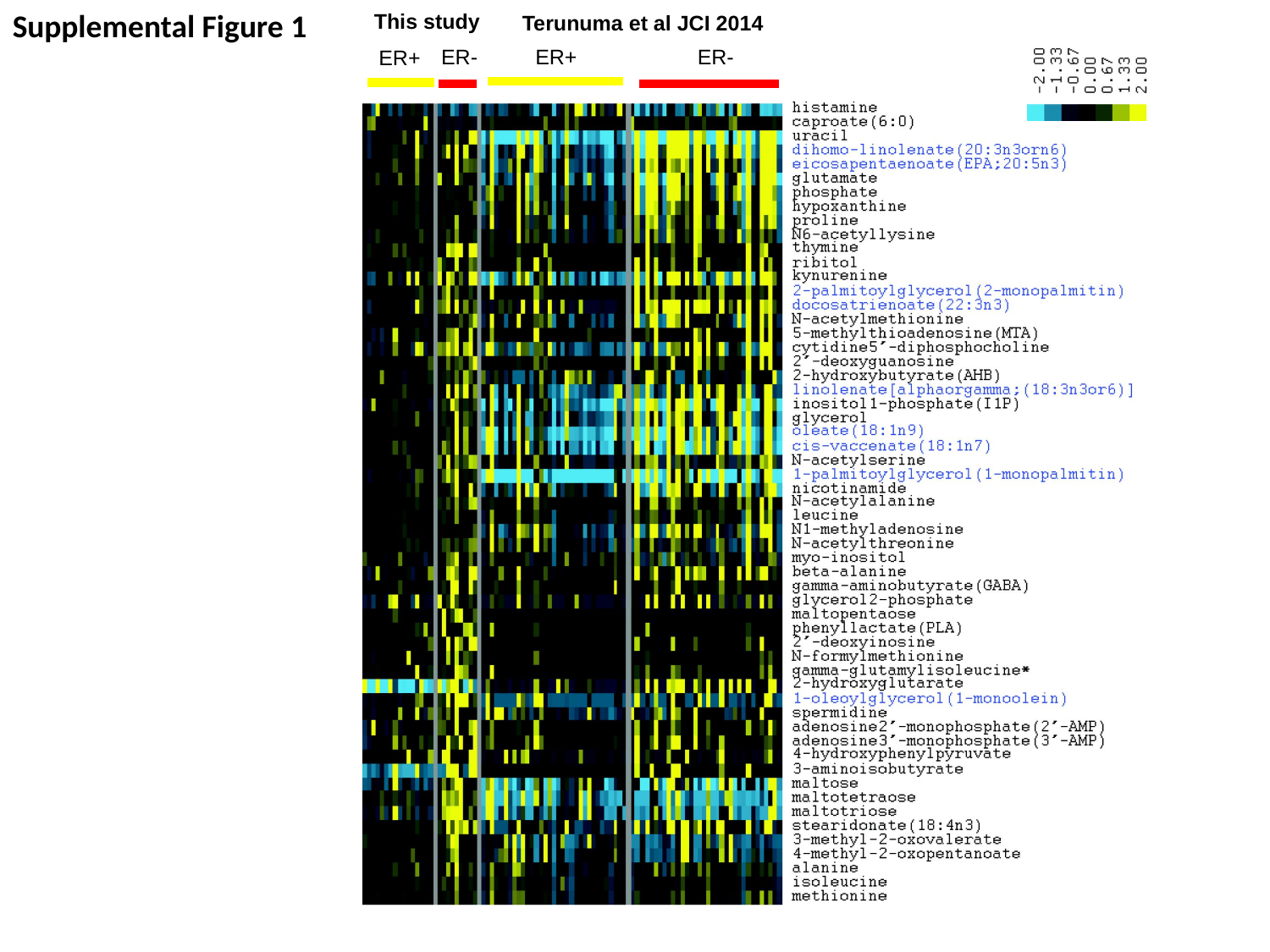

Supplemental Figure 1
This study
Terunuma et al JCI 2014
ER+
ER-
ER-
ER+

## Slide 2
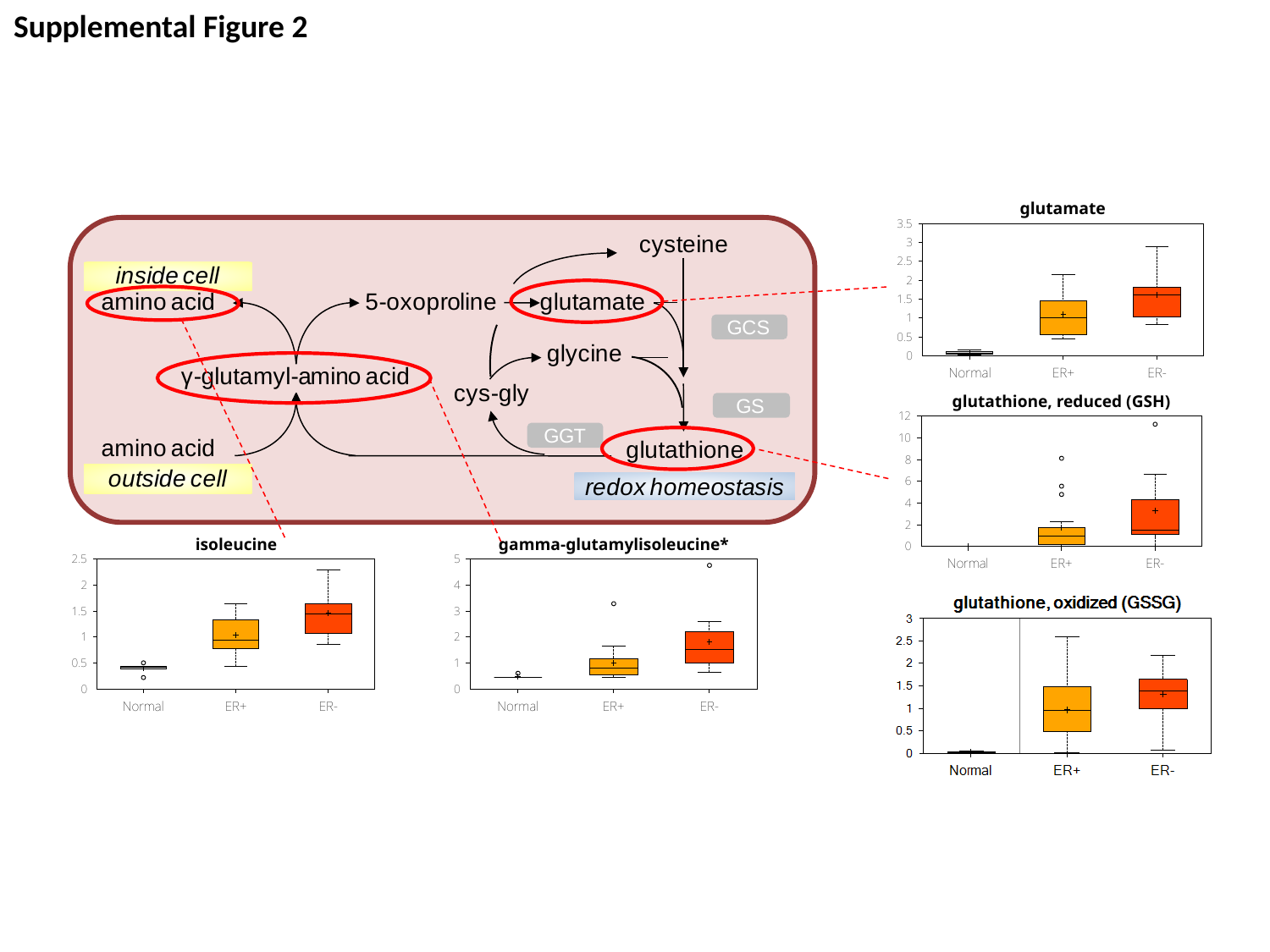

Supplemental Figure 2

## Slide 3
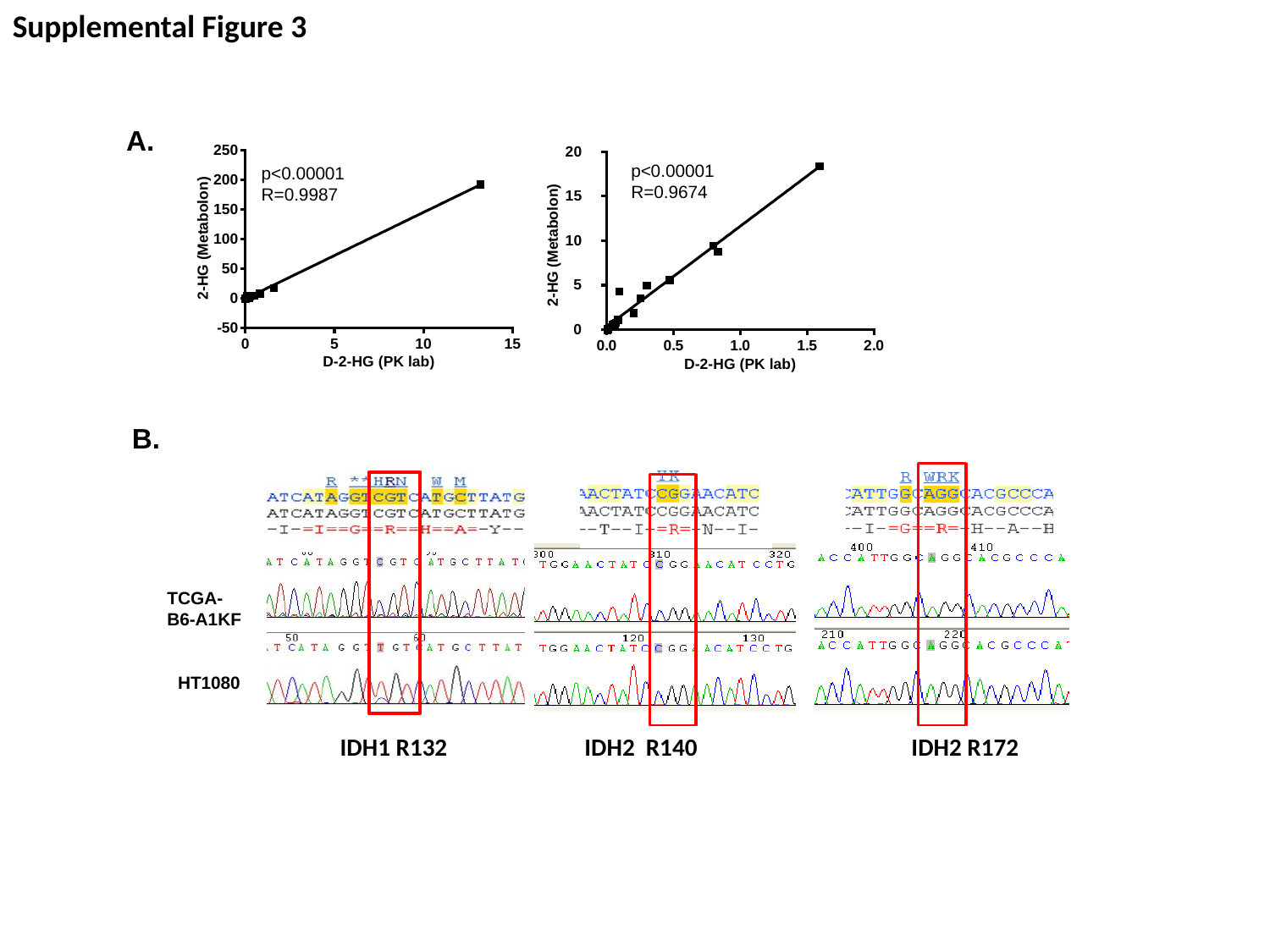

Supplemental Figure 3
A.
p<0.00001
R=0.9674
p<0.00001
R=0.9987
B.
TCGA-
B6-A1KF
HT1080
IDH1 R132 IDH2 R140 IDH2 R172

## Slide 4
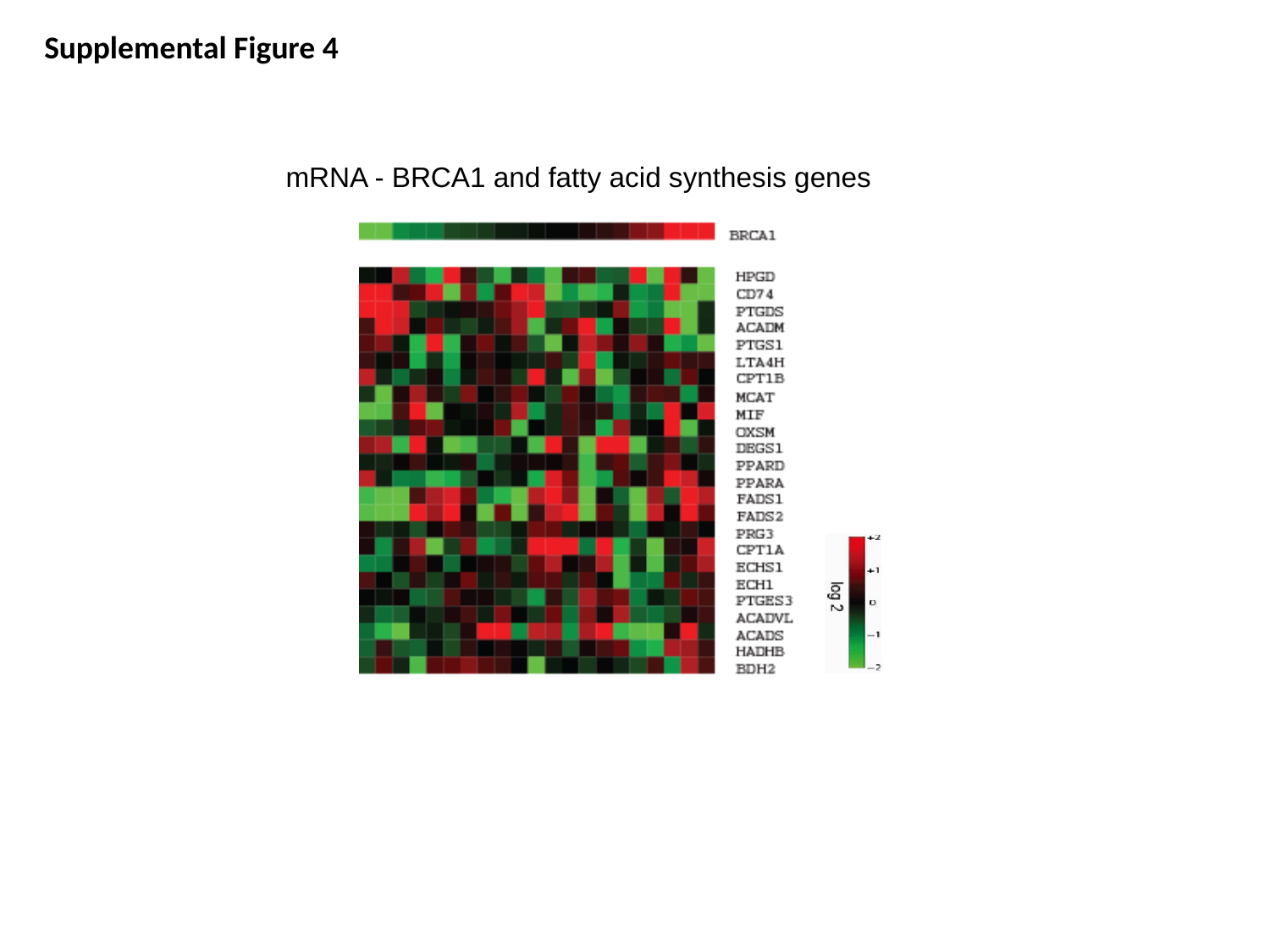

Supplemental Figure 4
mRNA - BRCA1 and fatty acid synthesis genes

## Slide 5
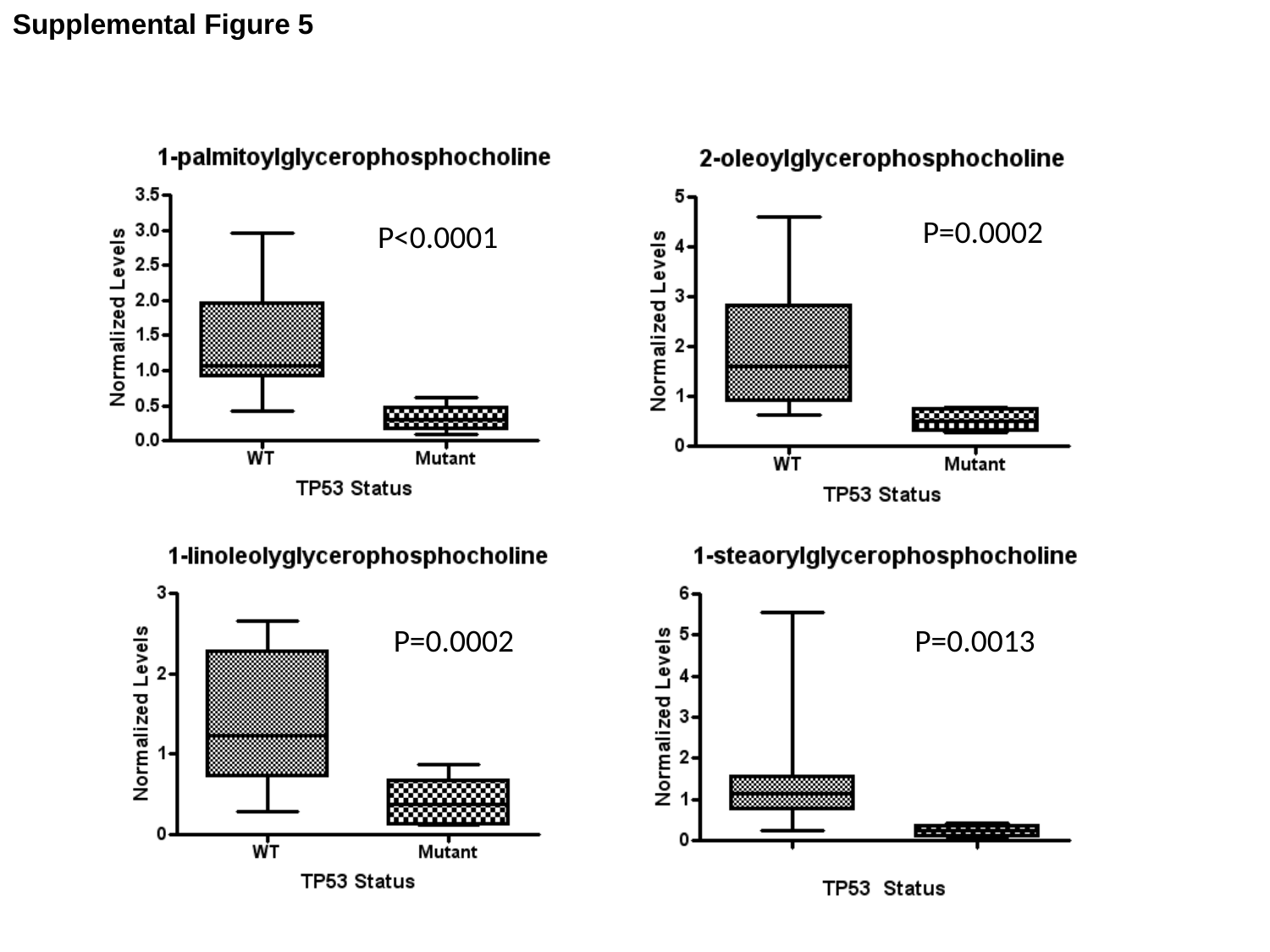

Supplemental Figure 5
P=0.0002
P<0.0001
P=0.0002
P=0.0013

## Slide 6
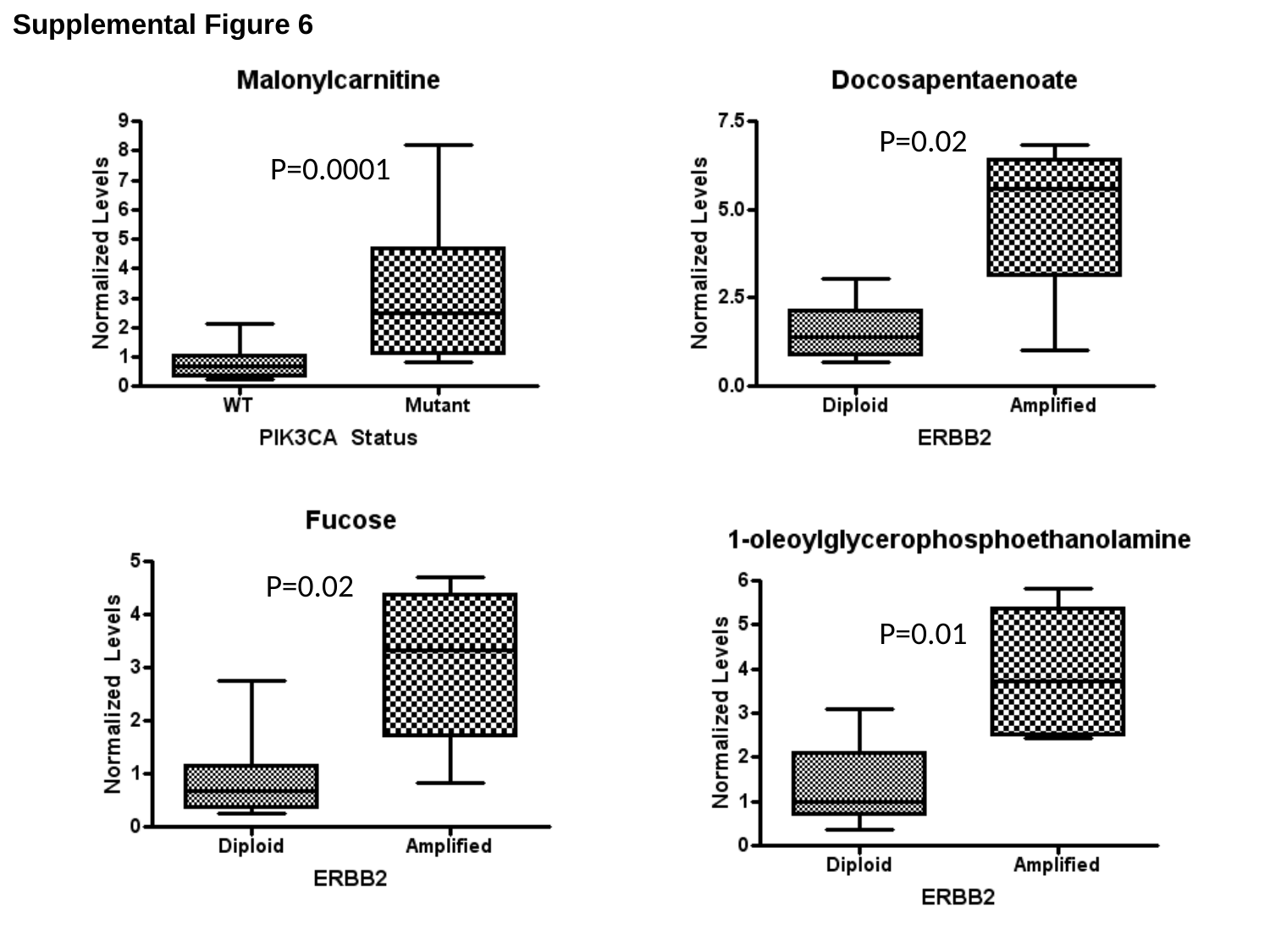

Supplemental Figure 6
P=0.02
P=0.0001
P=0.02
P=0.01

## Slide 7
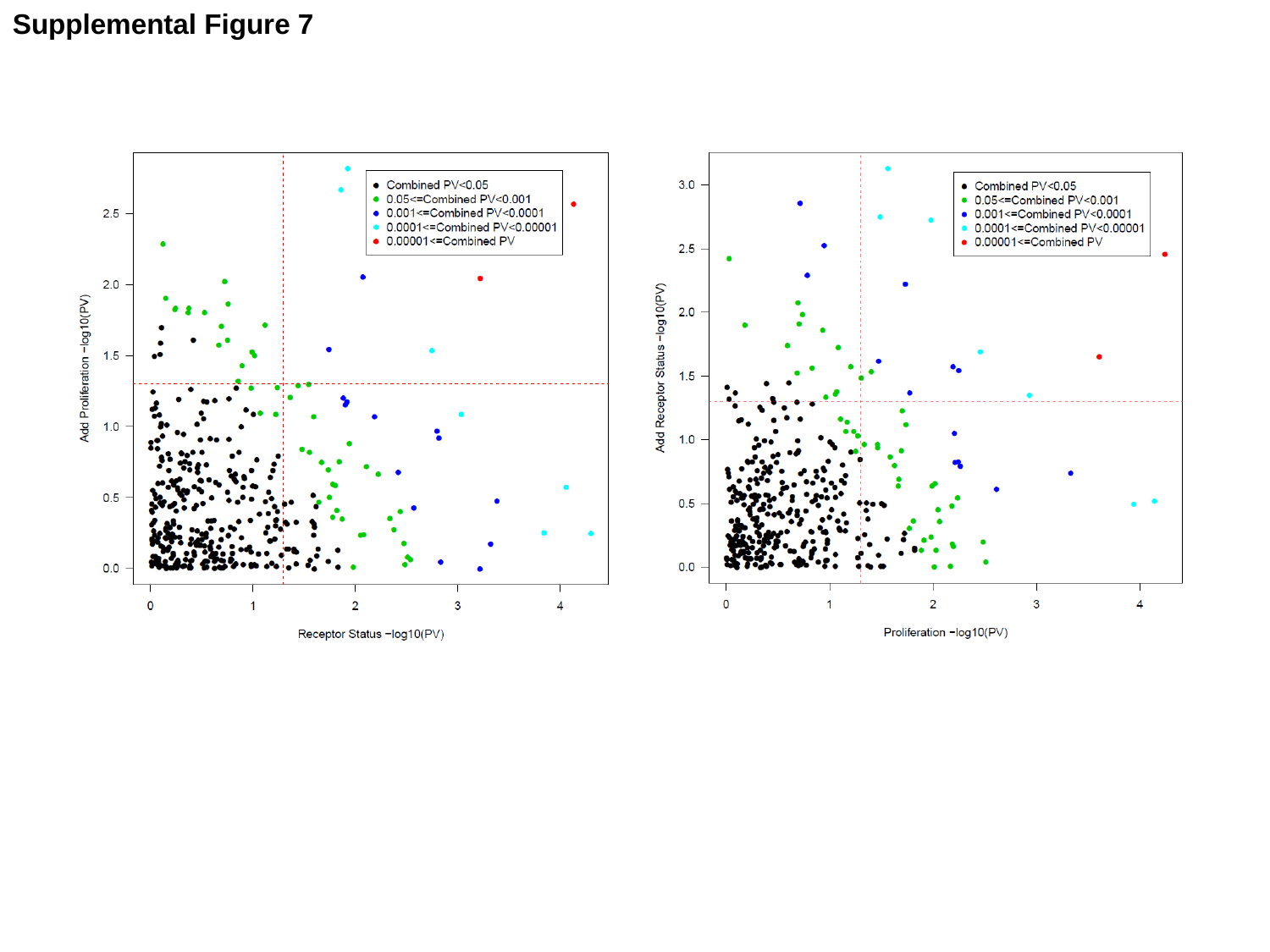

Supplemental Figure 7

## Slide 8
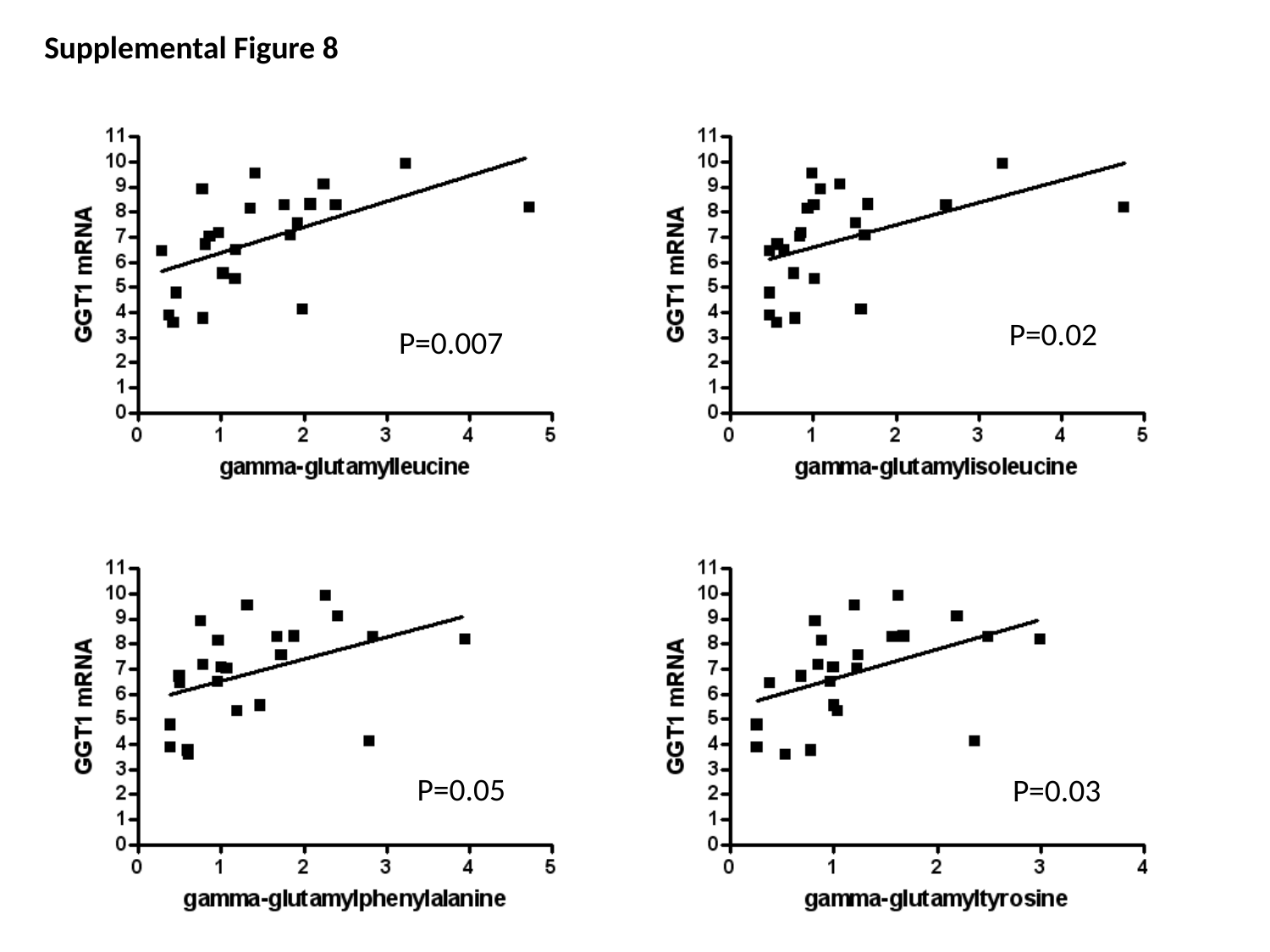

Supplemental Figure 8
P=0.02
P=0.007
P=0.05
P=0.03
