# Supplementary figures and images for: A joint analysis of metabolomics and genetics of breast cancer
Source: Breast Cancer Res. 2014 Aug 5;16:415. doi: 10.1186/s13058-014-0415-9 (PMC4187326; doi:10.1186/s13058-014-0415-9)

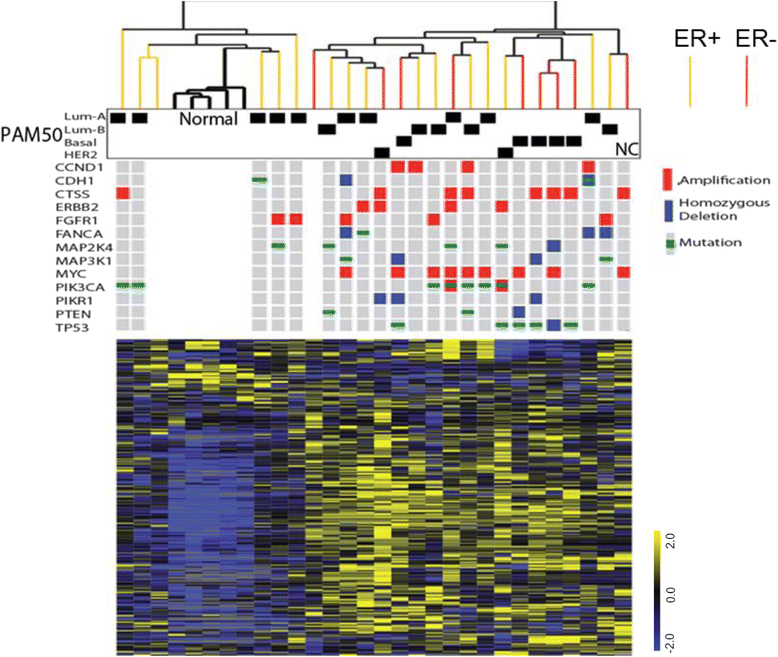

Supplement: Supplementary file 11 — Authors’ original file for figure 1 [file 13058_2014_415_MOESM11_ESM.gif]

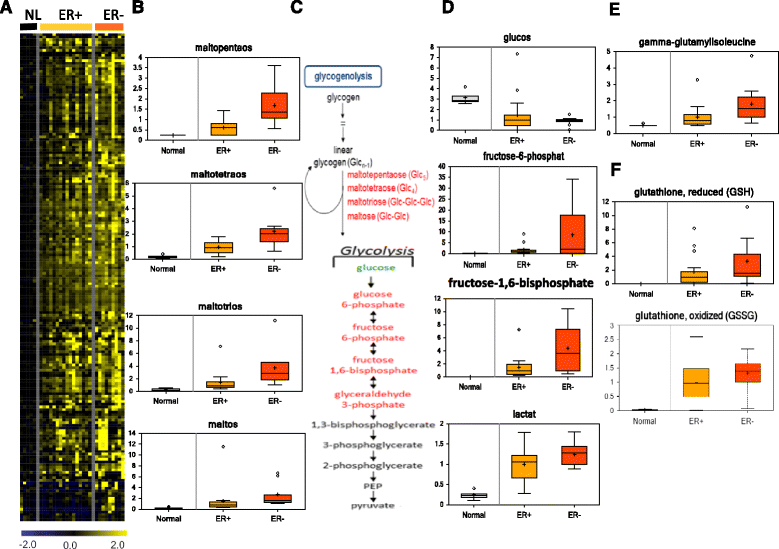

Supplement: Supplementary file 12 — Authors’ original file for figure 2 [file 13058_2014_415_MOESM12_ESM.gif]

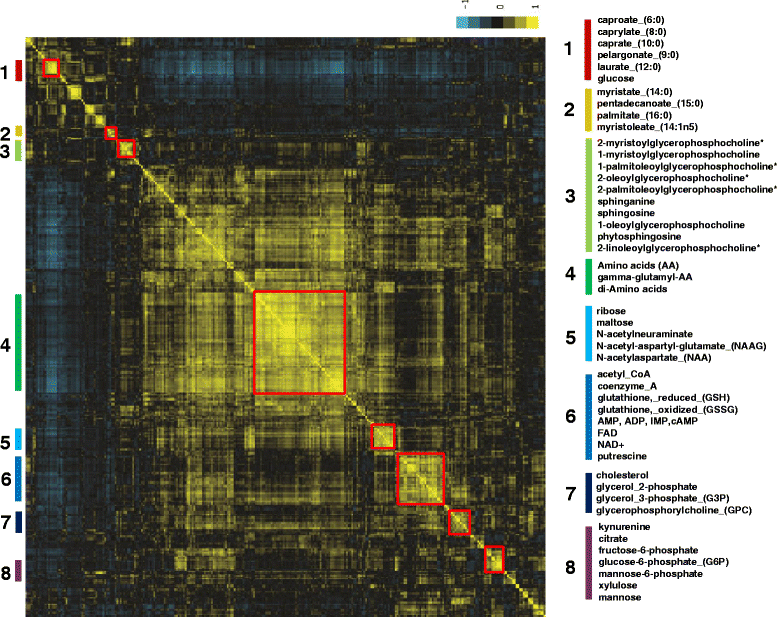

Supplement: Supplementary file 13 — Authors’ original file for figure 3 [file 13058_2014_415_MOESM13_ESM.gif]

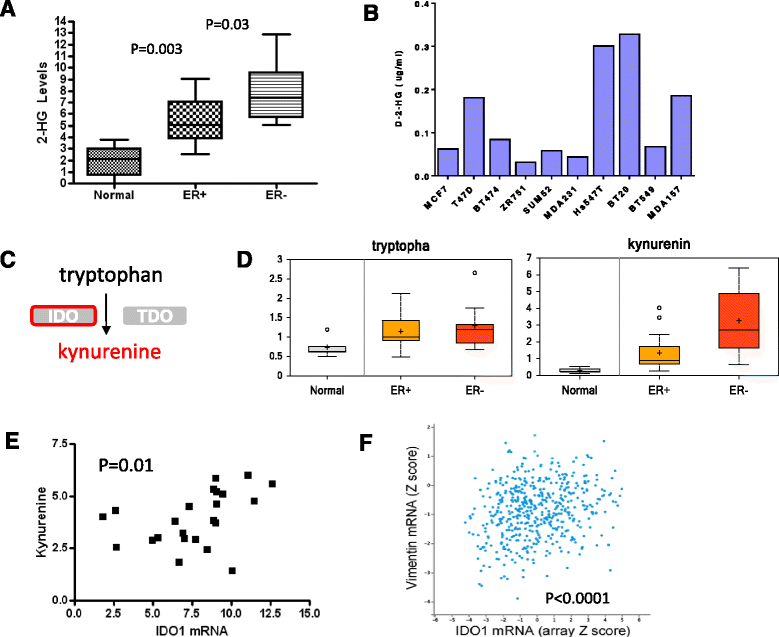

Supplement: Supplementary file 14 — Authors’ original file for figure 4 [file 13058_2014_415_MOESM14_ESM.gif]

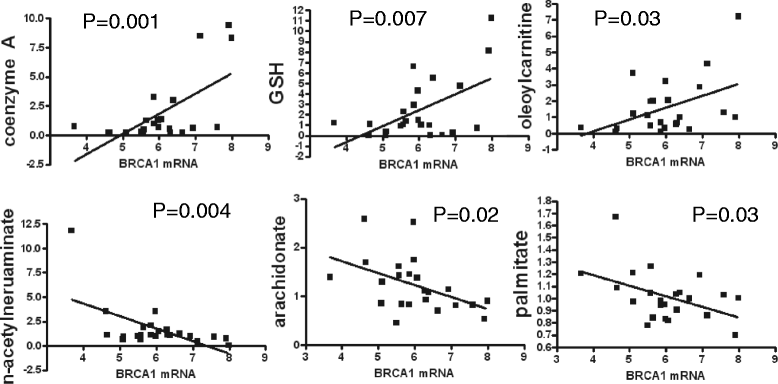

Supplement: Supplementary file 15 — Authors’ original file for figure 5 [file 13058_2014_415_MOESM15_ESM.gif]

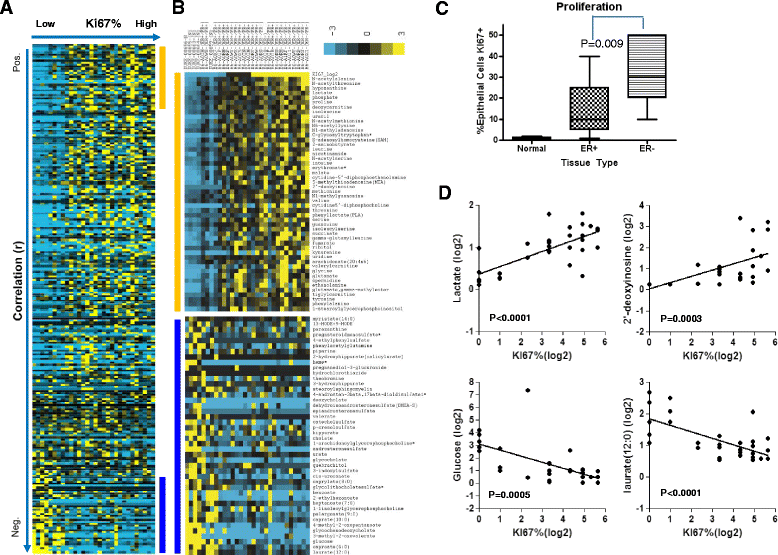

Supplement: Supplementary file 16 — Authors’ original file for figure 6 [file 13058_2014_415_MOESM16_ESM.gif]
